# Integration or separation in the processing of facial properties - a computational view

**DOI:** 10.1038/srep20247

**Published:** 2016-02-02

**Authors:** Christoph D. Dahl, Malte J. Rasch, Isabelle Bülthoff, Chien-Chung Chen

**Affiliations:** 1 Department of Psychology, National Taiwan University, Roosevelt Road, Taipei 106, Taiwan; 2 Department of Comparative Cognition, Institute of Biology, University of Neuchâtel, 2000, Rue Emile-Argand 11, Neuchâtel, Switzerland; 3 State Key Laboratory of Cognitive Neuroscience and Learning and IDG/McGovern Institute for Brain Research, Beijing Normal University, Xinjiekouwai Street 19, 100875 Beijing, China; 4 Max Planck Institute for Biological Cybernetics, Human Perception, Cognition and Action, Spemannstrasse 38, 72074 Tübingen, Germany

## Abstract

A face recognition system ought to read out information about the identity, facial expression and invariant properties of faces, such as sex and race. A current debate is whether separate neural units in the brain deal with these face properties individually or whether a single neural unit processes in parallel all aspects of faces. While the focus of studies has been directed toward the processing of identity and facial expression, little research exists on the processing of invariant aspects of faces. In a theoretical framework we tested whether a system can deal with identity in combination with sex, race or facial expression using the same underlying mechanism. We used dimension reduction to describe how the representational face space organizes face properties when trained on different aspects of faces. When trained to learn identities, the system not only successfully recognized identities, but also was immediately able to classify sex and race, suggesting that no additional system for the processing of invariant properties is needed. However, training on identity was insufficient for the recognition of facial expressions and vice versa. We provide a theoretical approach on the interconnection of invariant facial properties and the separation of variant and invariant facial properties.

Biological face perception systems deal with a multitude of facial properties: identity, facial expression and invariant properties of faces, such as sex and race. How the visual system deals with such immense amount of information is accounted for by models of visual systems^1^,^2^,^3^,^4^. The common denominator of these models is a design principle that independently processes facial properties in dedicated functional pathways. This architectural principle is further backed by neurophysiological findings^5^,^6^,^7^,^8^. However, recent evidence that the facial expression system consists of identity-dependent representations–among identity-independent ones–challenges the view of dedicated functional representations^9^,^10^. Such findings are further supported by early single-cell studies, revealing a subsample of neurons that responded to facial expressions as well as identities^6^,^11^. While the focus in most studies lies in investigating the processing characteristics of variant facial properties, like facial expression, and the invariant property, such as ‘identity’, it remains largely unaddressed whether invariant properties, like identity, sex and race, share processing. In this study, using a computational model we test (1) whether facial expression and identity are independent or, as recent literature suggests, interact to some degree and (2) in what manner combinations of invariant facial properties are processed. To disentangle these underlying principles, which the visual system uses to deal with variant and invariant aspects of faces, we followed a simple logic: We trained an algorithm (Linear Fisher Discriminant Analysis (LFD)) on one facial property (e.g. sex) and tested the algorithm on either the same facial property or on a different one (e.g. identity). This results in comparisons between (a) invariant facial properties only, (b) a combination of invariant and variant facial properties and (c) variant facial properties only. We conceive identity, sex and race as invariant and facial expression as variant facial properties. In brief, we labeled face images according to the face property in question, e.g. for identity it is a distinct label for each individual in the database. We then computed a number of linear fisher components, which maximize the variance between class examples and minimize the variance among examples with the same class labels. After training the components on one facial property, we relabeled the examples according to another face property and subsequently test classification performance of the new face property. If the performance on the new face property is high, one can assume that the face samples in the face space were organized by the first property well enough to support the processing of the second face property without the need of any reorganization. Thus a high performance of the second face property would support the view of a single neural unit dealing with both properties simultaneously.

## Results

**Figure 1:**
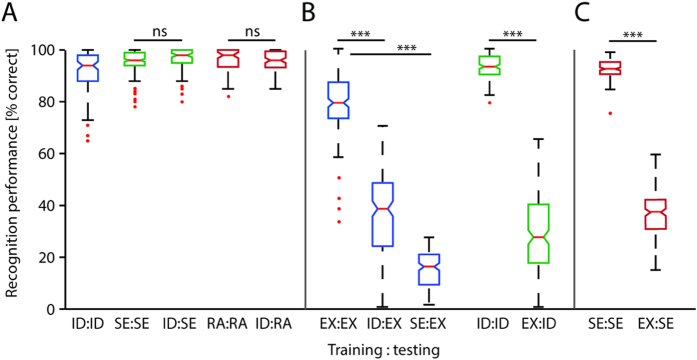
Recognition performances using Linear Fisher Discriminant Analysis. Identity trained Fisherface projections were applied to identity (ID:ID), sex (ID:SE), race (ID:RA) and facial expression (ID:EX); facial expression trained Fisherface projections were applied to facial expression (EX:EX), identity (EX:ID) and sex (EX:SE); sex trained Fisherface projections were applied to sex (SE:SE) and facial expression (SE:EX). (**A**) Percent correct classification for identity, sex and race properties. Color-codes of the boxplots refer to the facial properties tested; i.e. blue = identity, green = sex, red = race. (**B**) Percent correct classification for facial expression and identity properties. Color-codes of the boxplots refer to the facial properties tested; i.e. blue = facial expression, green = identity. (**C**). Percent correct classification for sex and facial expression properties. Color-codes of the boxplots refer to the facial properties tested; i.e. red = sex. (**A–C)**. Notches in boxplots indicate whether medians (red horizontal bars) are significantly different from each other. Non-overlapping notch intervals are significant at the 5% level. Whisker intervals cover +/−2.7 standard deviations (i.e. 99.3% in normally distributed data).

We found that when trained on identity of faces, the system performed well when tested on identity as expected (ID:ID, Fig. 1A, blue). The mean performance score is 91.91% (8.24% sd). The system achieved even better performances when trained and tested on sex (SE:SE, mean = 95.47%, sd = 4.4%, Fig. 1A, green) or race (RA:RA, mean = 96.05%, sd = 4.65%, Fig. 1A, red). The scores of the identity task (ID:ID) were significantly lower than those of the sex (SE:SE) (ID:ID vs SE:SE; *t*(283) = −4.75, *p *< 0.001) and race (RA:RA) tasks (ID:ID vs RA:RA; *t*(283) = −3.91, *p *< 0.001), which might simply rely on the fact that the identity task has a much greater number of distractors (i.e. the number of face identities) than the sex and race tasks. Interestingly, when the same system, trained on identity, was tested on sex (ID:SE) and race (ID:RA) its high performance levels were maintained (Fig. 1A, green and red). Sex classification (ID:SE) ranged at 96.29% correct classification (sd = 4.27%) and was not significantly different from the sex baseline condition (SE:SE) (SE:SE vs ID:SE; *t*(283) = −1.49, *p* = .14). Similarly, race classification (ID:RA) ranged at 95.69% correct classification (sd = 4.12%) and was not significantly different from the race baseline condition (RA:RA) (RA:RA vs ID:RA; *t*(283) = 0.53, *p* = 0.59). The absent of a performance deterioration from training and testing on the same invariant face properties to training and testing on different invariant face properties indicates that invariant features such as identity and sex, as well as identity and race, are processed in an integrative fashion.

We then trained and tested the system on facial expressions (EX:EX) and obtained an average level of performance at 77.7% correct classification (sd = 12.32%) (Fig. 1B, blue). Please note that the face stimuli used in this and the following comparisons were from a different database than above (see Methods). Training the system on identity and testing on facial expressions (ID:EX) resulted in an average performance level of 35.94% correct classification (sd = 16.03%, Fig. 1B, blue), and, thus in a significant deterioration as opposed to the baseline condition (EX:EX) (EX:EX vs ID:EX; *t*(340) = 27.01, *p *< 0.001). Further, when trained on sex and tested on facial expression (SE:EX) the system produced 16.45% correct classification (sd = 4.92%, Fig. 1B, blue) and significantly deviated from the baseline conditions (EX:EX) (EX:EX vs SE:EX; *t*(340) = 76.14, *p *< 0.001).

In a further step, we trained and tested the system on identity (ID:ID) and obtained an average of 94.69% correct classification (sd = 5.9%) (Fig. 1B, green). When training the system on facial expression and testing on identity (EX:ID) the performance scores deteriorated significantly relative to the baseline (ID:ID) (ID:ID vs EX:ID; *t*(340) = 50.88, *p *< 0.001; EX:ID mean = 27.17%, sd = 16.31%, Fig. 1B, green). Likewise we trained and tested the system on sex (SE:SE) and obtained an average of 93.54% correct classification (sd = 3.37%, Fig. 1C). We then trained the system on facial expression and tested it on sex (EX:SE). Under this condition the performance scores dropped to an average of 37.71% correct classification (sd = 10.31%, Fig. 1C) and, hence, was significantly different from the base line condition (SE:SE) (SE:SE vs EX:SE; ; *t*(340) = 70.21, *p *< 0.001).

Such deteriorative performance scores for cross-class conditions (ID:EX, SE:EX, EX:ID, EX:SE) as opposed to same-class conditions (EX:EX, ID:ID, SE:SE) indicate that facial expression and identity/sex are processed in a non-integrative way to a great extent.

**Figure 2:**
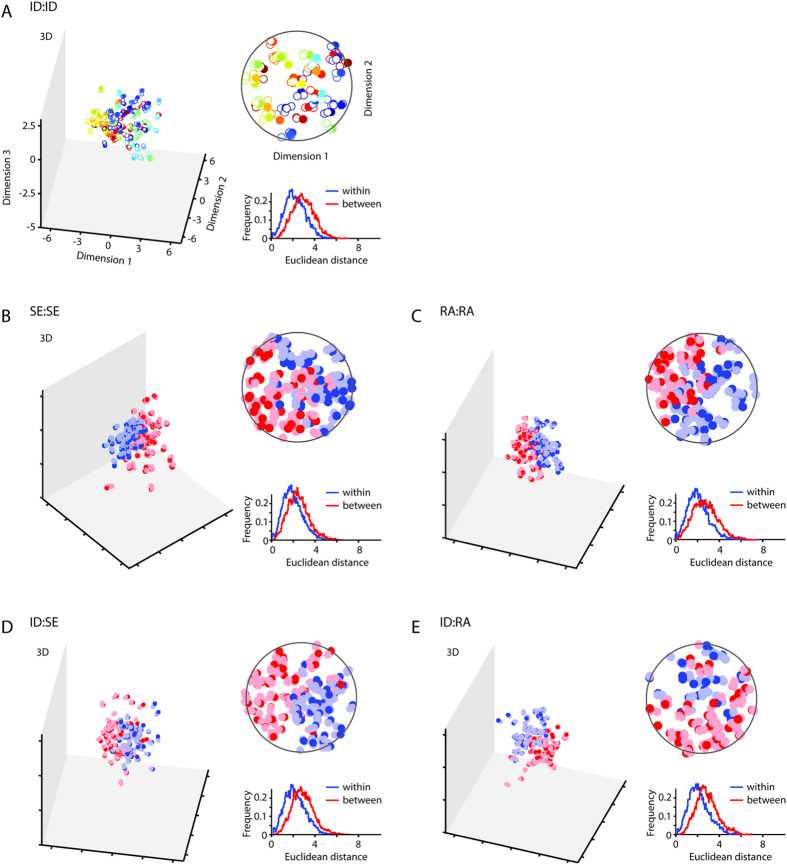
Face space for invariant facial properties. Fisherface projections of the first components. Each panel (**A**–**E**) shows the 3D projection of the first three components (left), and enlargement of the 2D projection of the first two components (upper right panel, magnifier plots) and a histogram of Euclidean distances of each sample to each other sample in the face space average according to class labels (‘within’: same class; ‘across’: difference classes). Figure titles indicate training and testing samples (e.g. training on identity and testing on race (ID:RA)). Colors in the 3D and 2D panels indicate classes. Note that in panel (**A**) due to the great number of classes (here identities), two different classes (identities) can have relatively similar colors but are located apart from each other in the face space. In panel (**A**) full circles indicate testing samples; outline circles indicate training samples. In (**B–E**) light colors indicate training samples.

**Figure 3:**
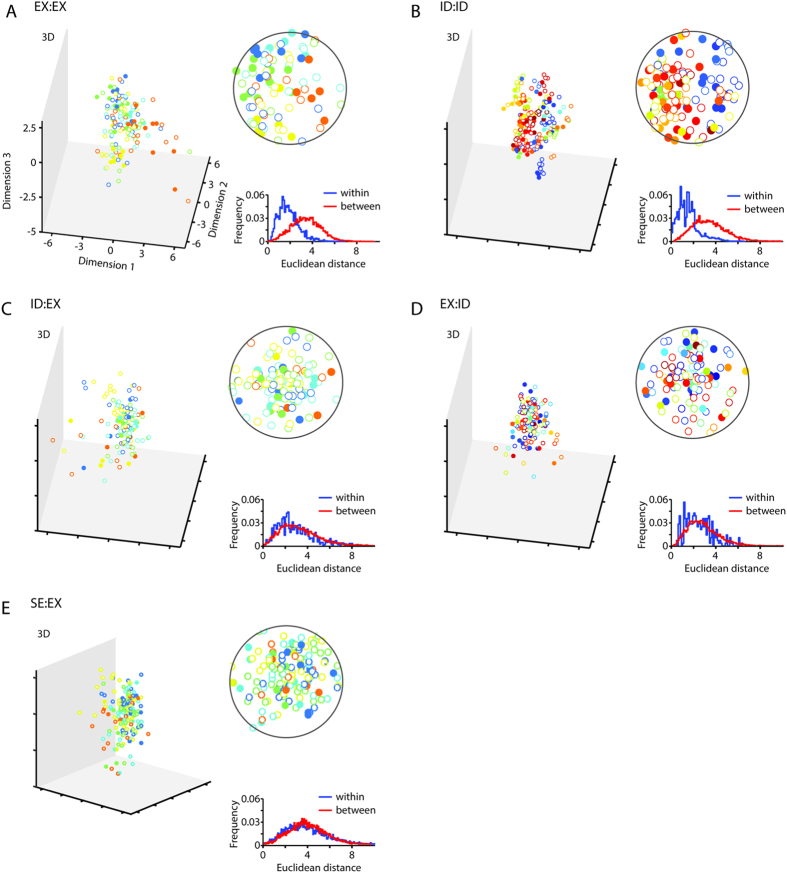
Face space for variant (facial expressions) and invariant facial properties (identity). Figure specifications as in Fig. 2.

**Figure 4:**
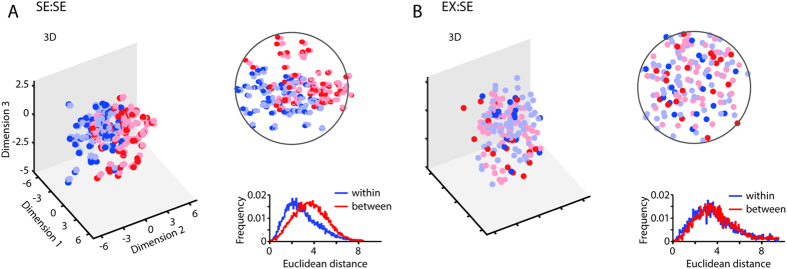
Face space for variant (facial expression) and invariant facial properties (sex). Figure specifications as in Figs. 2 and 3.

To get a more detailed view of the resulting embedding of the face properties into the faces space, we further investigated the underlying representational structures for sex and race, embedded in a set of identity-trained faces, and compared them to the resulting representational structures within the face space optimized for distinguishing facial expressions. To show the difference between the representations, we display the projections of samples in the respective Fisherface space. Figures 2, 3, 4 show the projections of the first components and the histograms of average Euclidean distances between and within classes. Classes are color-coded. In an optimized system, members of the same class are expected in close proximity to each other. It can be seen that the amount of clustering into corresponding classes is equally high for the conditions involving training and testing on the same labels (ID:ID, Fig. 2A; SE:SE, Fig. 2B; RA:RA, Fig. 2C) as well as for conditions involving invariant facial features (ID:SE, Fig. 2D; ID:RA, Fig. 2E), reflected in ‘Within’ distances being smaller than ‘Between’ distances (see histograms: ID:ID: *D* = 0.28, *p *< 0.001, Fig. 2A; SE:SE: *D* = 0.23, *p *< 0.001, Fig. 2B; RA:RA: *D* = 0.25, *p *< 0.001, Fig. 2C; ID:SE: *D* = 0.26, *p *< 0.001, Fig. 2E; ID:RA: *D* = 0.32, *p *< 0.001, Fig. 2F). The fact that cross-class conditions (ID:SE and ID:RA) show identical representational patterns as same-class conditions (SE:SE and RA:RA) illustrates that an intrinsic structure in the representation of sex and race properties evolved when building up the face space along identity properties. On the other hand, there is a dissociation for the conditions involving both variant and invariant facial properties: The amount of clustering is higher for same-class conditions (e.g. EX:EX, ID:ID; Fig. 3A,B), than for cross-class conditions (ID:EX, EX:ID, SE:EX; Fig. 3C–E). ‘Within’ distances are smaller than ‘Between’ distances in the conditions EX:EX (*D* = 0.58, *p *< 0.001, Fig. 3A) and ID:ID (*D* = 0.63, *p *< 0.001, Fig. 3B), not in the conditions ID:EX (*D* = 0.01, *p* = 0.99, Fig. 3C), EX:ID (*D* = 0.001, *p* = 0.81, Fig. 3D) and SE:EX (*D* = 0.01, *p* = 0.99, Fig. 3E). Likewise, ‘Within’ distances are smaller than ‘Between’ distances in the same-class condition for sex (SE:SE) (*D* = 0.75, *p *< 001, Fig. 4A), but they are equidistant in the cross-class condition (EX:SE) (*D* = 0.01, *p *< 0.99, Fig. 4B).

The fact that cross-class conditions (ID:EX, EX:ID, SE:EX, EX:SE) show differential representational patterns as same-class conditions (EX:EX, ID:ID, SE:SE) illustrates that information from variant facial properties are not intrinsically represented when building up the face space along invariant facial properties and vice versa.

## Discussion

The aim of the study was to investigate the dependency, or independency, of variant and invariant facial properties. It would be premature to assume that the brain incorporates any of the algorithms used in our simulation. However, such implementation serves a statistical analog of the representation of faces^3^,^12^. Further, the model does not allow any deduction of underlying neural principles. It provides insight into the nature of information processing of facial properties for various classification attributes. We trained algorithms on facial identity, sex, race, and expression and tested each of them on properties of the identical class (ID:ID, SE:SE; RA:RA, EX:EX) or of the non-identical classes (ID:SE, ID:RA; ID:EX, SE:EX, EX:ID, EX:SE). We found that, in the testing phase, classification scores of sex and race were high, irrespective of whether the algorithm has been trained on the identical classes (race and sex, respectively) or a non-identical class (identity). In contrast, performance scores deteriorated drastically when the algorithm was trained on identity and tested on facial expression and vice versa. A similar deterioration of performance scores was found when training the algorithm on sex and testing it on facial expression and vice versa. Together, these findings support the notion that invariant facial properties do not require separate independent processing, but can be dealt with in one and the same underlying processing module to a great extent. The data further supports the notion of independent processing of identity and facial expression, as well as sex and facial expression, by showing strong deteriorative effects when these classes of facial properties are approached in one processing step.

Invariant facial properties: A recent study by Zhao and Hayward^13^ showed that sex and race influenced the process of identity analysis: Variations in sex or race affected the identification of a face, indicating that the processing of identity is co-dependent on the processing of sex or race. With our study, we provide a computational account on these findings by providing insights into the representational structure of faces. Together, the idea of co-dependent processing of invariant facial features is inconsistent with the model by Bruce and Young^1^,^4^,^14^. In their model, sex and race were assumed to provide semantic information about a face, putting them automatically into qualities with separate processing. Haxby and colleagues^2^ proposed independent brain networks for variant and invariant facial features. Invariant facial properties (like identity, sex and race) were located within the lateral fusiform gyrus (e.g. FFA) and recruit similar neural correlates. As predicted, the fusiform gyrus is not only involved in the processing of identity, but largely shares neural correlate associated with the processing of sex and race^15^,^16^,^17^,^18^. E.g., Ng *et al*.^17^ showed a network of activated brain structures for identity, gender and race (ethnicity) that spread across inferior occipital cortex, the fusiform gyrus and the cingulate gyrus, suggesting that certain dimensions of facial features for identity, gender and race are processed in similar neural substrate and, hence, in co-dependency. Further, analyzing event-related potentials (ERP) and magnetic fields produced by the electric currents occurring in the brain (MEG), face sex selective responses were found at 100–150 ms after stimulus onset^19^,^20^,^21^,^22^, suggesting little or no top-down processing, such as visual attention^23^. In addition, as shown by^22^, intentional sex discrimination affected the ERPs starting from 200 ms and ending at 250 ms. Here, we showed that with semi-supervised classification algorithms, automatic sex and race information is retrieved. Such automatic processing was the byproduct of identity classification. Interestingly, sex and race processing fall onto an overlapping timeframe with implicit identity processing (individuation of faces) around 120 to 190 ms^24^,^25^. At later more explicit stages (around 250 ms) sex and race selective timeframes overlap with memory-related activation of faces as well as the integrative process of face and voice^26^. Hence, there is evidence that (a) sex and race information is automatically acquired with the analysis of identity^13^, (b) neural substrates in the face responsive network of the brain code identity, sex and race in overlapping fashion^15^,^16^,^18^, and (c) computational algorithms for dimensionality reduction produces a representational structure, explicitly (with training) representing identities and implicitly (without training) representing sex and race, as shown in the current study. It is important to note that identity, sex and race are conceptual descriptions that contain a number of dimensions on which relevant information is coded. Hence, the fact that the model classifies faces on sex and race successfully, after having been trained on identity, only indicate that *certain* dimensions are shared, not necessarily all. Additional support for this comes from a computational study using autoassociative neural networks^27^. The purpose of this study was to account for the other-race effect (ORE^28^) by the multidimensional facespace^29^. As expected, the facespaces for same-race faces was wider than the facespaces for other-races faces, irrespective of whether Caucasian or Asian faces were used in the learning phase. Besides that the similarity between adjacent faces was higher in same-race than other-race facespaces and neighboring samples appeared to be of the same sex. Even though there was no identity learning involved, the spontaneous organization of face representations is intriguingly similar to the results presented in our study.

Variant facial properties: According to the model of Haxby *et al*.^2^, a two-stream separation of face processing into variant facial aspects (facial expression) in the superior temporal sulcus (STS) and invariant facial aspects (identity, sex, race) in the lateral fusiform gyrus follows early face representations in occipitotemporal cortex. The assumption about neurologically independent systems was addressed by a number of psychological studies. The dissociation of two pathways comes from following findings (see^3^ for detailed description): (a) Imaging studies revealed dissociable neural representations of identity and facial expression^30^,^31^. (b) Monkey studies showed selective responses of cell populations to either identity or facial expression^6^. (c) Familiarity of faces does not affect identification of facial expressions and vice versa^32^,^33^. (d) Brain-injured patients were selectively impaired for either identity or facial expression^34^,^35^. (e) Previous approaches with linearized compact coding schemes explained the partial separability of identity and facial expression^12^,^36^. In sum, these studies are supportive for a relative segregation of identity and facial expression information processing. On the other hand, there is growing evidence that facial expression information is not completely independent of identity information^3^. Single-cell recordings in the monkey revealed that a subsample of neurons was responsive to facial expressions as well as identities^6^,^11^. Interactions between neural correlates of facial expression and identity have been pinpointed in more recent single-cell recordings^37^,^38^,^39^ and imaging studies^40^,^41^. These relatively small numbers of neurons have been found in the superior temporal sulcus^6^,^37^,^38^, the inferior temporal gyrus^6^ and the amygdala^39^. The latter receives inputs from both the superior temporal sulcus and the inferotemporal cortex and might play a role in the integration of facial expression and identity^42^. Further, numerous behavioral studies using an adaptation paradigm have demonstrated that aftereffects in facial expressions are modulated by the identity of the target and adapting faces, i.e., aftereffect for same-identity conditions were larger than those for different-identity conditions^10^,^43^,^44^. In contrast, the representation of identity is independent of changes in facial expression^45^, i.e., the aftereffect of identity was not affected by whether or not the target and adapting face were of the same expression. Together, given the small proportion of neurons responsive for both facial expression and identity and the unidirectional nature of dependency, an adequate description of the processing of facial expression and identity is between a strict separation and a complete unity. Our data shows that there is little shared among the facial features that allow classification across representations of facial expression and identity.

In the framework proposed, we embed faces in an optimally designed face space under rules of energy constraints. We term it the Space Constrained Optimized Representational Embedding (SCORE) approach^46^. As it becomes obvious in Figs 2, 3, 4, identity and sex, or race, cluster according to spatial rules with exemplars of the same identity in minimal distances from each other and exemplars of different identities with maximal distances from each other. Embedded in this trained structure of optimal allocation of identities in space, an implicit and fully untrained substructure of sex or race emerged at the level of the first two dimensions. Hence, dimensionality reduction leads to the most critical and diagnostic dimensions explaining morphological variations in faces, such as sex and race. Sex and race are semantic categories^1^, or in other words concepts that are fundamental to humans to live and act in their environment. Interestingly, at a theoretical level (and restricted to faces), implementing these concepts happens intrinsically and automatically. Sex and race become systematically embedded in the face space for identity.

From an evolutionary point of view it is not only critical to successfully classify what can be eaten, what is dangerous, who is dominant^47^, or what I can use as a tool^48^, but rather who is friend or foe–a question relying on successful within class discrimination^49^,^50^,^51^,^52^,^53^,^54^,^55^, or in other words subordinate-level classification^56^. While the “what is that” questions might be sufficient for survival, the “who is that” questions can only be addressed in a functionally relevant face classification system^57^. Connectionist network models have stimulated the emergence of category representations^58^,^59^, as described in infants^60^, and found a trend of learning more global category representation prior to more distinct categorical grouping (global-to-basic-to-subordinate representations^61^). In somewhat similar fashion, using facial stimuli as inputs to such models, we would predict the emergence of the most differentiated categorization scheme, sex or race, first, followed by subordinate level or individuation (‘person A’, ‘mom’, ‘Silvia’)^62^. Our findings indicate that the system acquired information at both levels of classification by approaching the concepts at the lowest level of class inclusion and lowest degree of generality. A global-to-basic-to-subordinate learning curve suggests that while the infant is exposed to an increasing number of faces, the structural development of representation will be systematically differentiated and optimized over months and years to achieve best results at the subordinate level of classification. This assumption is in accordance with findings in child development of face processing^63^,^64^. Further, an integrative approach of basic and subordinate level classification in one system gives a handle on tracking changes of representation with increasing amount of visual expertise in a class that ultimately helps explain effects like the other-race effect^27^,^65^, the other-species effect^66^, as well as the mirror-effect^46^,^67^.

## Material and Methods

### General

To test our hypotheses we ran a computer simulation based on a regular PC using Matlab (Mathworks Inc., Natick, MA, USA).

### Stimuli

We used rendered 1445 images from 3-D reconstructions of faces^38^,^39^, consisting of faces of 1060 Caucasians and 385 Asians of both sexes from five viewpoints each (–10, 0, 10, 20 and 30 degrees). We selected randomly 120 face identities that were equally split across sex and race. These face images were taken from the face database of the Max Planck Institute for Biological Cybernetics. These faces were used for the following conditions: ID:ID, SE:SE, ID:SE, RA:RA, ID:RA, as in Fig. 1A. A second set of images contained the Taiwanese Facial Expression Image Database (TFEID), established by the Brain Mapping Laboratory (National Yang-Ming University) and Integrated Brain Research Unit (Taipei Veterans General Hospital), and our own face database^68^. These databases consist of photographs of 50 individuals with six facial expressions (neutral, anger, fear, happiness, sadness, surprise). Half of the individuals were female, all of them were Asians. The images were photographs that included outer facial features such as hairline. The viewpoint was frontal (+/–0 degree). These faces were used in the following conditions: EX:EX, ID:EX, SE:EX, ID:ID, EX:ID, SE:SE, EX:SE. All face images were gray-scaled. Due to privacy rights, we do not show face stimuli, but refer to the online database for face samples (http://faces.kyb.tuebingen.mpg.de). We did not include any other face database for cross-validation, e.g. training with faces from one database and testing with faces from another database.

### Linear Fisher Discriminant (LFD)

We assume that the neural machinery of face processing has access to the complex non-linear feature space of faces, representing the facial features as extracted from high-dimensional face space via sensory processing. Neural resources are limited and representational embedding of features has to be optimized. One way of representing such complex data is to find a subspace which represents most of the face variance. We first reduced the data complexity by using Principal Components Analysis (PCA), in the context of faces, yielding a set of Eigenfaces. These Eigenfaces can be described as the eigenvectors of the largest eigenvalues of the covariance matrix of the training face data set. Face images were of identical pixel resolution (90 × 90). Each face image was treated as one vector of concatenated rows. The mean of all faces was subtracted from all images. The eigenvectors and eigenvalues of the covariance are then calculated. Since all eigenvectors contain the same dimensionality as the original face images, they can be considered a face image, thus called Eigenfaces, and reflect the deviation from the mean face. We then applied a discriminant analysis, known as the Linear Fisher Discriminant (LFD), which chooses a subspace that best maps sample vectors of the same class in minimal distances and sample vectors of different classes in maximal distances (by calculating sample variances between classes S_B_ and within classes, S_W_, and then solving a generalized Eigenvector problem, for mathematical details see^69^). As a result, we find a new reduced set of vectors with the same dimensionality as the Eigenvectors above, where the original face can be projected to. These projections are called Fisherfaces in the literature^69^. Before computing the Fisherfaces, we preprocess the original face images by applying a PCA to reduce dimensionality (to n = 10). The Fisher-faces are computed based on the class labels of the training set (e.g. expression, or identity, or sex). Note that these Fisherfaces are different when the class label set of the train set differs (e.g. Fisherfaces trained on identity are in general different from that of Fisherfaces trained on sex). When the test class label set was different from the “train” label test set, we first projected the face sample of the Fisherfaces generated from the training label set and then re-labeled the resulting vectors and finally tested classification performance of that projected sample. Note that when the Fisherfaces for identity would not have some inherent information about sex the sex information would be randomly distributed in the Identity-Fisher-space and thus classification performance would be poor. Note that the number of independent dimensions of the LFD subspace cannot be larger than the number of classes minus 1, see^69^. Thus, for our comparison of the Fisher-faces for different number of classes, we added random orthogonal dimensions to the subspace where necessary.

To estimate the classification performance on the projected sample, we employed a simple distance based approach similar to a delayed matching-to-sample task (DMS) in psychophysical experiments^70^. We first randomly selected two faces from the data set, each corresponding to a distinct class. We then randomly chose a test face example from one of the two classes and judged the trial correct if the test sample had nearer Euclidean distance in the projected face-space to the face of the same class, otherwise incorrect. We iterated this procedure over all available pairs of faces and calculated the percentage correct trials. Note that if faces of the same class are clustered very nearby in face space and very far from other classes, performance will be very high.

### Data analysis

The analyses were performed using Matlab (Mathworks Inc., Natick, MA, USA). The dependent variables were percentage correct classification and Euclidean distances in the representational space. Percentage correct responses were compared across conditions using two-sample t-tests (Fig. 1). The data samples for each condition contained performance values derived from test runs which varied in the number of components [6:2:42] and face samples [20:10:120] or [20:10:160]. Data was omitted if the number of face samples was smaller than the number of components. For the Fisherface spaces in Figs 2, 3, 4 we used the following number of faces: 120 for the Max Planck Institute database (conditions: ID:ID, SE:SE, RA:RA, ID:SE, ID:RA) and 160 for the TFEID database (including our own database) (conditions: EX:EX, ID:ID, SE:SE, EX:ID, ID:EX, SE:EX, EX:SE). Histograms (Euclidean distances), as shown in Figs 2, 3, 4, were compared using one-sided (‘Between’ > ‘Within’) two-sample Kolmogorov-Smirnov tests.

## Additional Information

**How to cite this article**: Dahl, C. D. *et al*. Integration or separation in the processing of facial properties - a computational view. *Sci. Rep.* **6**, 20247; doi: 10.1038/srep20247 (2016).
